# Synthesis, Structural Characterization, Antimicrobial Activity, and In Vitro Biocompatibility of New Unsaturated Carboxylate Complexes with 2,2′-Bipyridine

**DOI:** 10.3390/molecules23010157

**Published:** 2018-01-12

**Authors:** Gina Vasile Scăețeanu, Mariana Carmen Chifiriuc, Coralia Bleotu, Crina Kamerzan, Luminiţa Măruţescu, Constantin G. Daniliuc, Cătălin Maxim, Larisa Calu, Rodica Olar, Mihaela Badea

**Affiliations:** 1 Department of Soil Sciences, University of Agronomical Sciences and Veterinary Medicine, 59 Mărăşti Str., Sector 1, 011464 Bucharest, Romania; ginavasile2000@yahoo.com; 2 Department of Microbiology, Faculty of Biology, University of Bucharest, 1–3 Aleea Portocalelor Str., 60101 Bucharest, Romania; carmen_balotescu@yahoo.com (M.C.C.); crina.saviuc@yahoo.com (C.K.); lumi.marutescu@gmail.com (L.M.); 3 Research Institute of the University of Bucharest–ICUB, Life, Environmental and Earth Sciences Division, Spl. Independentei 91–95, 010271 Bucharest, Romania; 4 Stefan S. Nicolau Institute of Virology, 285 Mihai Bravu Ave., 030304 Bucharest, Romania; cbleotu@yahoo.com; 5 SC Sanimed International Impex SRL, Sos Bucuresti Magurele, nr. 70F, Sector 5, 051434 Bucharest, Romania; 6 Organisch-Chemisches Institut, Westfälische Wilhelms-Universität Münster, Corrensstrasse 40, 48149 Münster, Germany; constantin.daniliuc@uni-muenster.de; 7 Department of Inorganic Chemistry, Faculty of Chemistry, University of Bucharest, 90–92 Panduri Str., 050663 Bucharest, Romania; maxim_catalin@yahoo.com (C.M.); larisa.calu@yahoo.com (L.C.)

**Keywords:** 2,2′-bipyridine complexes, acrylato ligand, trinuclear complex, antifungal activity

## Abstract

The synthesis, structural characterization, cytotoxicity, and antimicrobial properties of four new complexes formed by employing acrylate anion and 2,2′-bipyridine are reported herein. X-ray crystallography revealed the trinuclear nature of [Mn_3_(2,2′-bipy)_2_(C_3_H_3_O_2_)_6_] (**1**), meanwhile complexes with general formula [M(2,2′-bipy)(C_3_H_3_O_2_)_2_(H_2_O)*_x_*]∙*y*H_2_O ((**2**) M: Ni, *x* = 1, *y* = 0; (**3**) M: Cu, *x* = 1, *y* = 0; (**4**) M: Zn, *x* = 0, *y* = 1; 2,2′-bipy: 2,2′-bipyridine; C_3_H_3_O_2_: acrylate anion) were shown to be mononuclear. The lowest minimum inhibitory concentration (MIC) of 128 μg mL^−1^ was recorded for all four tested complexes against *Candida albicans*, for complex (**3**) against *Escherichia coli*, and for complex (**4**) against *Staphylocococcus aureus*. Compounds (**3**) and (**4**) were also potent efflux pumps activity inhibitors (EPI), proving their potential for use in synergistic combinations with antibiotics. Complexes (**1**)–(**4**) revealed that they were not cytotoxic to HCT-8 cells. They also proved to interfere with the cellular cycle of tumour HCT-8 cells by increasing the number of cells found in the S and G2/M phases. Taken together, these results demonstrate the potential of zinc and copper complexes for use in the development of novel antimicrobial and anti-proliferative agents.

## 1. Introduction

Carboxylate complexes have an extremely rich chemistry, due mostly to various coordination modes exhibited by carboxylate groups [1] and to the presence of various coligands found in the coordination sphere of the metallic ions [2,3,4].

The use of unsaturated carboxylates as ligands has increased tremendously lately due to their interesting structural architectures [3,5] and biologic properties [6,7].

If unsaturated carboxylates possess polymerisable groups, they become suitable for biological applications (tissue engineering, implantable medical devices, dentistry, bone repair, etc.) due to their low molecular weight, compositions, and architectures that may be modulated through controlled reactions [8,9].

A thorough analysis of already published papers reveals that acrylic acid (an O-donor anionic ligand) has been used in the synthesis of complexes together with different nitrogen donor ligands, such as 2,2′-bipyridine [10,11], 4,4′-bipyridine [12], 1,10-phenantroline [11,13,14,15], ethylenediamine [16], imidazole [17,18], pyrazole derivatives [19], benzimidazole derivatives [20,21,22,23,24], and triazole [25], or oxygen donors such as trimethylphosphate [26] and urea [27].

The interest in acrylate and its derivative complexes arises due to their fluorescent [13], antibacterial [11,28], and antitumor properties [29], and antiferomagnetic interactions [12,26,30].

The emergence of multi-drug, extended-drug, and pan-drug resistance among bacterial and fungal pathogens is presently a global health problem urging the development of novel antimicrobial agents, either from natural sources or obtained by chemical synthesis [31].

In this paper we report on the synthesis, spectral features, structural characterization, antimicrobial activity, and in vitro biocompatibility studies of some new complexes with mixed ligands. The manganese compound is a trinuclear species [Mn_3_(2,2′-bipy)_2_(C_3_H_3_O_2_)_6_] (**1**), while the other complexes are mononuclear species with the general formula: [M(2,2′-bipy)(C_3_H_3_O_2_)_2_(H_2_O)*_x_*]∙*y*H_2_O ((**2**) M: Ni, *x* = 1, *y* = 0; (**3**) M: Cu, *x* = 1, *y* = 0; (**4**) M: Zn, *x* = 0, *y* = 1; 2,2′-bipy: 2,2′-bipyridine; C_3_H_3_O_2_: acrylate anion). The crystal structures for complexes (**1**)–(**4**) have been solved. Preliminary data regarding the thermal behavior of these kinds of complexes has already been reported [10].

## 2. Results and Discussion

### 2.1. Synthesis of the Complexes

In this paper we report the synthesis, structural characterization, and bioevaluation of four new complexes containing mixed ligands, i.e., the acrylate ion and 2,2′-bipyridine. The complexes’ formulae have been established on the basis of chemical analysis, IR spectra, and X-ray diffraction analysis as follows:

[Mn_3_(2,2′-bipy)_2_(C_3_H_3_O_2_)_6_] (**1**)

[Ni(2,2′-bipy)(C_3_H_3_O_2_)_2_(H_2_O)] (**2**)

[Cu(2,2′-bipy)(C_3_H_3_O_2_)_2_(H_2_O)] (**3**)

[Zn(2,2′-bipy)(C_3_H_3_O_2_)_2_]·H_2_O (**4**)

where 2,2′-bipy is 2,2′-bipyridine and C_3_H_3_O_2_ is the acrylate anion.

All complexes were obtained in two steps: firstly, metallic acrylates were obtained using raw materials such as carbonates or oxides; the second step consisted of the reaction of the metallic acrylates with 2,2′-bipyridine.

### 2.2. Characterization of the Complexes

#### 2.2.1. Description of the X-ray Crystal Structures of the Complexes

A summary of the crystallographic data and structure refinement for crystals (**1**)–(**4**) is given in Table 1.

X-ray Structure of Complex [Mn_3_(2,2′-bipy)_2_(C_3_H_3_O_2_)_6_] (**1**)

The trinuclear linear compound [Mn_3_(2,2′-bipy)_2_(C_3_H_3_O_2_)_6_] is framed in structural prototype presented in literature [Mn_3_(RCOO)_6_(N–N)_2_], where:

(a) RCOO^−^ is the anion of isobutyrate and N–N is 1,10-phenantroline/2,2′-bipyridine [32];
(b) RCOO^−^ is the acetate ion, while N–N is 2,2′-bipyridine [33], 1,10-phenantroline [34], 2,2′-bis(1-methylimidazolyl)-phenylmetoxymethane [35], 2-(2-pyridyl)benzimidazole [36], *N*-(1,3-dimethylimidazolidin-2-ylidene)quinolin-8-amine [37];
(c) RCOO^−^ is chloroacetate and N–N is 2,2′-bipyridine [38].
(d) RCOO^−^ is the cinnamate ion and N–N is 2,2′-bipyridine [39]

Other studies evidenced that trinuclear linear manganese complexes with neutral bidentate nitrogen ligands and acetate may possess catalytic activity and selectivity in the epoxidation of olefins under mild conditions [40]. Having in view this proven behavior for this type of complex, further studies concerning this aspect will be discussed in a subsequent paper.

**Figure 1:**
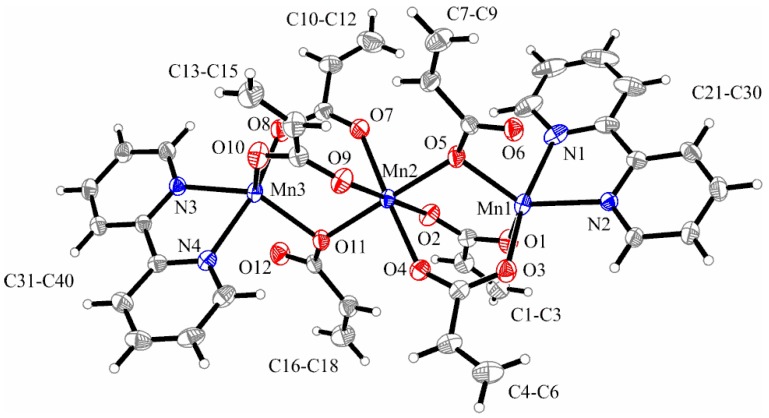
X-ray structure of [Mn_3_(2,2′-bipy)_2_(C_3_H_3_O_2_)_6_] (**1**). Thermal ellipsoids are shown with 30% probability.

As depicted in Figure 1, carboxylate ions adopt various coordination modes, i.e., bridge through two oxygen atoms, monoatomic bridge, and chelate. This coordination behavior of carboxylate ligands is encountered mainly in trinuclear linear complexes similar to this one [32,37,38,39,41].

Bond distances and angles for the coordination spheres are collected in Table 2.

In the trinuclear complex with linear Mn arrangement, each pair of manganese atoms is bridged by three acrylato ligands, two of them coordinated in a bidentate fashion, whereas the third bridging acrylate coordinates in a unidentate mode via O5 and O11, respectively. The central manganese (Mn2) has a more regular octahedral environment formed exclusively by six oxygen atoms of acrylate ions and is located on a crystallographic inversion center. Mn2–O distances range from 2.1613(15) to 2.1862(14) Å, while O–Mn2–O angles range from 88.50(6)° to 91.66(6)°.

The coordination environments for Mn1 and Mn3 can be described as distorted octahedral, each coordination polyhedron being comprised from two nitrogen atoms provided by 2,2′-bipyridine, two oxygen atoms from one acrylate ion that acts as chelate and monoatomic bridge simultaneously, and two oxygen atoms given by two bridged acrylate ions. As noticed from Table 2, Mn1–O and Mn3–O bonds are smaller than Mn2–O bonds, similar to those observed for manganese complexes with acetate [33] or chloroacetate [38], and 2,2′-bipyridine. No relevant π-π- interactions involving the bipyridine moieties were observed in the packing diagram.

**Figure 2:**
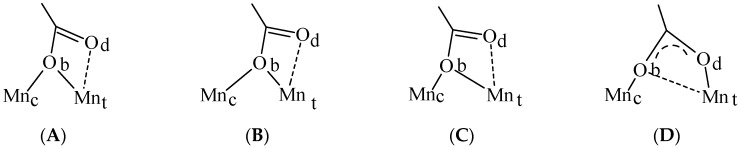
Carboxylate bridging mode in trinuclear complexes with the general formula [Mn_3_(RCOO)_6_(N–N)_2_] [42].

Gómez and Corbella [42] reported a systematic review of the structural parameters of trinuclear compounds of the type [Mn_3_(RCOO)_6_(N–N)_2_] containing carboxylate ligands which act simultaneously as chelate and unidentate bridge, and concluded that they can be arranged into four classes (structures A–D in Figure 2).

According to their systematization, complex (**1**) belongs to category B with an asymmetric unidentate bridge displaced towards the terminal manganese ion (similar with acetate and chloroacetate complexes, and unlike isobutyrate and cinnamate complexes which belong to category A, Table 3).

The reported Mn**_…_**Mn distances (3.4682(1) Å for Mn1**_…_**Mn2 and 3.4848(1) Å for Mn2**_…_**Mn3) are shorter than those reported in the cases of [Mn_3_(O_2_CCH_3_)_6_(bpy)_2_], [Mn_3_(O_2_CCH_2_Cl)_6_(bpy)_2_], and [Mn_3_(O_2_CCH(CH_3_)_2_)_6_(dpa)_2_]·2MeCN complexes (3.614, 3.624, and 3.611(4) Å, respectively) [33,38,43], and almost equal to those from analogous isobutyrate complexes with 2,2′-bipyridine (3.4894(3) Å) [32]. The Mn–N bond distances range from 2.2091(17) to 2.2670(2) Å, which is close to the values reported for manganese trinuclear complexes with 2,2′-bipyridine, acetate [33], 2-chloroacetate [38], and isobutyrate [32] ions.

The linear arrangement of the trinuclear compound is sustained by the Mn1–Mn2–Mn3 angle of 178.11(1)°.

X-ray Structure of Complex [Ni(2,2′-bipy)(C_3_H_3_O_2_)_2_(H_2_O)] (**2**)

**Figure 3:**
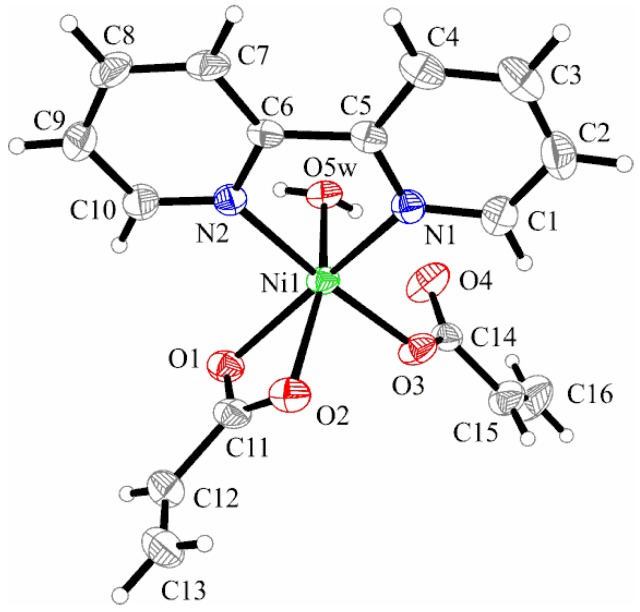
X-ray structure of [Ni(2,2′-bipy)(C_3_H_3_O_2_)_2_(H_2_O)] (**2**). Thermal ellipsoids are shown with 30% probability.

The crystal structure of compound (**2**) consists of neutral [Ni(2,2′-bipy)(C_3_H_3_O_2_)_2_(H_2_O)] complexes (Figure 3). The Ni(II) ion is six-coordinated by two bipyridine-nitrogen atoms, two oxygen atoms from one chelating acrylate, one oxygen atom from the other acrylate ion, and one oxygen from a coordinated water molecule, building a somewhat distorted octahedral surrounding.

**Figure 4:**
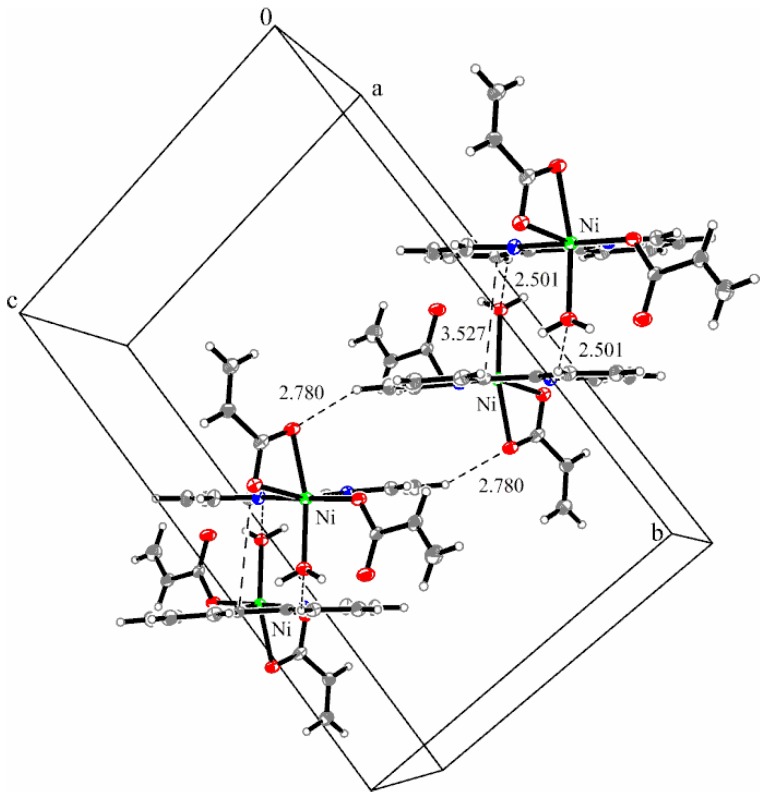
Packing diagram presenting the C–H**_…_**O hydrogen bonds and π**_…_**π interactions along the *a*-axis in complex (**2**).

The acrylato ligands adopt two different coordination modes: one acts unidentate (Ni1–O3 = 2.034(2) Å) and the other is chelate bidentate (Ni1–O1 = 2.126(2) Å, Ni1–O2 = 2.147(3) Å). X-ray analysis indicates that the ethylene group in the chelating acrylate ligand is disordered over two crystallographic positions with occupancies of 75% and 25%, respectively. The shortest distance between the aromatic rings of bipyridine is about 3.527 Å, indicating the presence of weak π**_…_**π–interactions. The packing diagram (Figure 4) presents the formation of dimers involving π**_…_**π–interactions and weak C–H**_…_**O hydrogen bonds between the bipyridine unit and the water oxygen atom (C4–H4**_…_**O5w 2.501 Å). These dimers are building linear chains along the *a*-axis through additional C–H**_…_**O hydrogen bonds between the chelate acrylato ligand and the vicinity bipyridine unit (C9–H9**_…_**O1 2.780 Å). 

X-ray Structure of Complex [Cu(2,2′-bipy)(C_3_H_3_O_2_)_2_(H_2_O)] (**3**)

**Figure 5:**
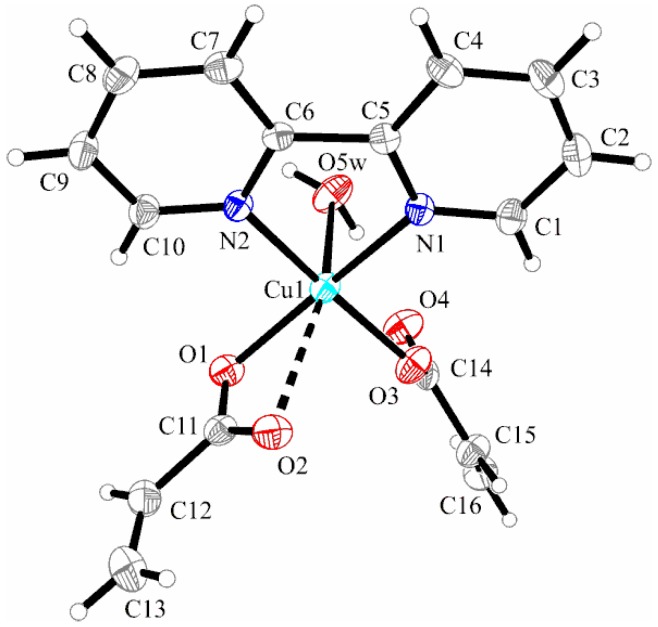
X-ray structure of [Cu(2,2′-bipy)(C_3_H_3_O_2_)_2_(H_2_O)] (**3**). Thermal ellipsoids are shown with 30% probability. (Only molecule “A” of the two found in the asymmetric unit is shown).

The asymmetric unit of complex (**3**) presents two crystallographic independent mononuclear entities of [Cu(2,2′-bipy)(C_3_H_3_O_2_)_2_(H_2_O)] (Figure 5). As the structural parameters are very similar, only molecule “A” is discussed below. Copper (II) ions are six-coordinated by two bipyridine-nitrogen atoms, two oxygen atoms from one chelating acrylate, one oxygen atom from a unidentate acrylato ion, and an oxygen atom from a coordinated water molecule, building a slightly distorted octahedral geometry.

The geometry of complex (**3**) is almost identical to that of complex (**2**), but the coordination modes adopted by the chelating acrylato ligand in compound (**3**) differ slightly. The chelating acrylato ligand is unsymmetrically bonded to the Cu atom [Cu–O1 1.991(3) Å and Cu–O2 2.637(3) Å]. The unidentate acrylato ligand (Cu–O3 1.949(3) Å), bipyridine ligand (Cu–N1 2.021(3) Å), and Cu–N2 (2.012(3) Å) present shorter bond lengths compared to the Ni complex, while the Cu–O_water_ bond is weaker (2.289(3) Å) than the Ni–O_water_ bond (2.060(3) Å).

**Figure 6:**
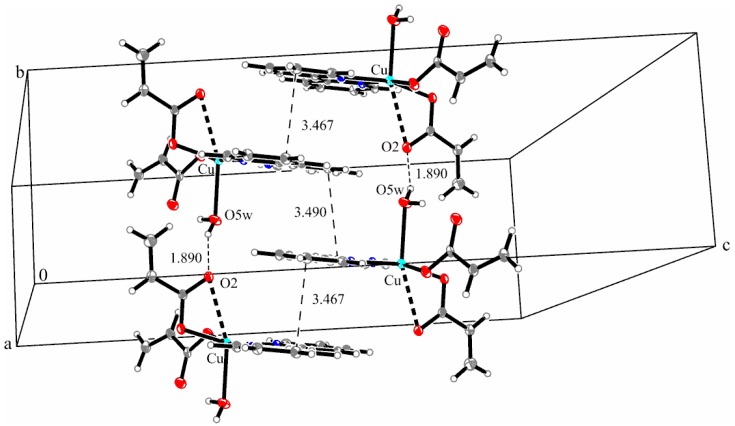
Packing diagram presenting the O–H**_…_**O hydrogen bond and π**_…_**π interactions along the *b*-axis in complex (**3**).

The presence of a coordinated water molecule and the acrylate coordination mode was alleged by IR spectra analysis. The distance between metallic ions in the asymmetric unit is 9.558 Å. The shortest distance between the aromatic rings of bipyridine is 3.467 Å, thus indicating π**_…_**π stacking interactions compared to those found for the Ni complex. The packing diagram of complex **3** (see Figure 6) is different from that found for compound **2** and presents the formation of linear chains along the *b*-axis involving strong O–H**_…_**O hydrogen bonds (O5W–H02**_…_**O2 1.890 Å) and π**_…_**π interactions with alternate distances (3.467 Å and 3.490 Å).

X-ray Structure of Complex [Zn(2,2′-bipy)(C_3_H_3_O_2_)_2_]·H_2_O (**4**)

Crystallographic investigation of complex (**4**) reveals a structure made up of two mononuclear units [Zn(2,2′-bipy)(C_3_H_3_O_2_)_2_] that are crystallographically independent in asymmetric cells.

**Figure 7:**
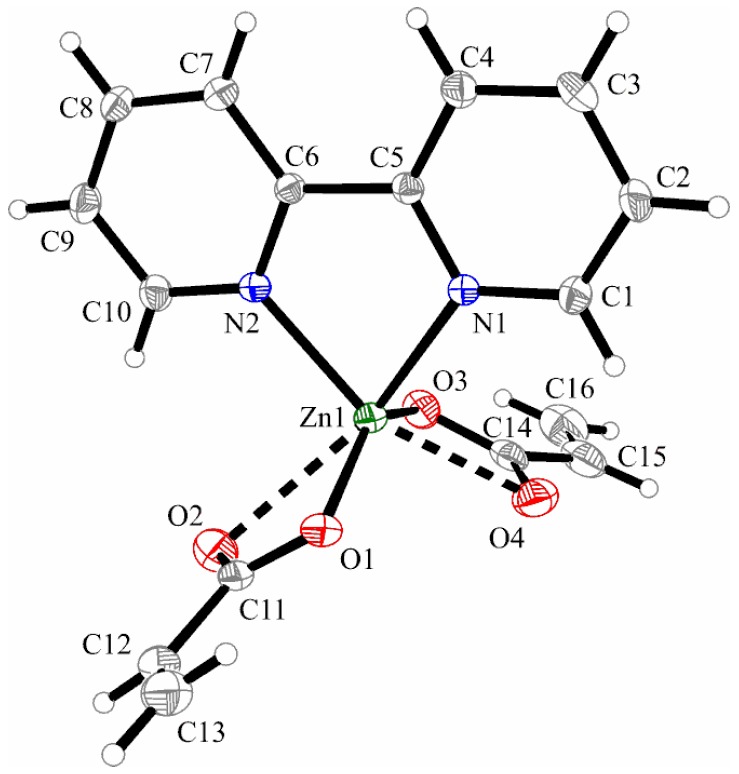
X-ray structure of [Zn(2,2′-bipy)(C_3_H_3_O_2_)_2_]·H_2_O (**4**). Thermal ellipsoids are shown with 15% probability. (Only molecule “A” of two found in the asymmetric unit is shown).

The metallic ion is six-coordinated by two bipyridine-nitrogen atoms and four oxygen atoms provided by two acrylate ions, building a distorted octahedral environment. Similar to the cooper complex (**3**), only the molecule “A” is discussed below. Both acrylato ligands adopt an asymmetrical bidentate coordination manner with two short Zn–O bond lengths (Zn1–O1 1.994(3) and Zn1–O3 2.032(3) Å) and two longer Zn–O bonds (Zn1–O2 2.490(3) and Zn–O4 2.326(3) Å). The Zn–N bonds from the bidentate 2,2′-bipyridine ligand are in the same range as the complexes with Ni and Cu ions (Zn1–N1 2.100(3) and Zn1–N2 2.099(2) Å) (Figure 7).

The bidentate coordination of acrylate was anticipated on IR spectra analysis, according to ∆ criterion [1,43].

**Figure 8:**
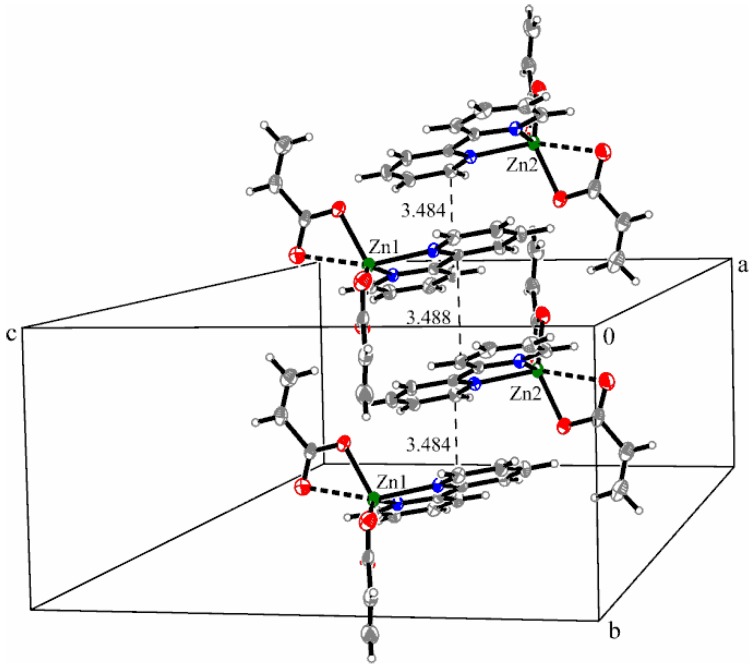
Packing diagram presenting the π…π interactions along the *b*-axis in complex (**4**).

Each acrylate ion participates at the equatorial plane with one oxygen atom, the other positions being occupied by nitrogen atoms. The apical positions are conquered by the remaining oxygen atoms of acrylate ions. The distance between metallic ions in the asymmetric unit is 7.987 Å. The shortest distance between the aromatic rings of bipyridine is 3.469 Å, indicating weaker π**_…_**π stacking interactions compared to the copper complex (Figure 8).

The main bond lengths and angles for mononuclear complexes (**2**)–(**4**) are listed in Table 4.

The values of M–N and M–O bond lengths observed for mononuclear complexes (**2**)–(**4**) fall in the range reported for similar compounds with mixed ligands: 2,2′-bipyridine/2,2′-bipyridine derivatives and carboxylato derivatives (Table 5) [44,45,46,47,48].

#### 2.2.2. Infrared Spectra

The bands that appear in IR spectra (Figure S1, Supplementary Materials) in the range 1535–1540 cm^−1^ are characteristic of 2,2′-bipyridine and are shifted in comparison with the free ligand, indicating the chelate coordination of aromatic ammine. The presence of acrylate anions in the composition of complexes is sustained by the appearance of ν_as_(COO) and ν_s_(COO) bands in the IR spectra of all complexes.

The splitting of bands ν_s_(COO) or ν_as_(COO) that appear in complexes (**1**) and (**2**), respectively, may be associated according to literature studies [1,49], with different coordination modes adopted by the carboxylate ligand. This statement was confirmed by X-ray structure in the case of complex (**2**).

Also, it is known that the ∆ value (∆ = ν_as_(COO) − ν_s_(COO)) indicates the coordination mode associated with carboxylate [1]. Therefore, for complex (**3**), a ∆ value of 211 cm^−1^ indicates a unidentate coordination for acrylate, meanwhile for (**4**) a value of 183 cm^−1^ suggests a bidentate coordination mode [49]; the latter statement is proven by X-ray analysis for (**3**).

The presence of water molecules in complexes (**2**)–(**4**) generates a large band that appears in the range 3350–3460 cm^−1^, and it is assigned to ν(OH) stretching vibrations [50]. Also, a medium intensity band that appears in the range 630–640 cm^−1^ for complexes (**2**) and (**3**) may be assigned to vibration mode *ρ*_w_(H_2_O) and suggests the coordinated nature of water molecules.

#### 2.2.3. Electronic Spectra

The diffuse electronic reflectance spectra recorded for Mn(II), Ni(II), and Cu(II) (Figure S2, Supplementary Materials) offered information regarding the coordination number and stereochemistry of complexes.

In the case of manganese complex [Mn(2,2′-bipy)(C_3_H_3_O_2_)_2_]∙H_2_O (**1**), the electronic spectrum reveals a band at 375 nm that can be assigned to the ^6^A_1g_ → ^4^T_2g_(D) spin-forbidden transition characteristic of d^5^ high spin octahedral species.

The electronic spectrum of [Ni(2,2′-bipy)(C_3_H_3_O_2_)_2_(H_2_O)] (**2**) presents three bands associated with d-d spin-allowed transitions for d^8^ high-spin octahedral species. The assignments and position of the bands are: ^3^A_2g_ → ^3^T_2g_ (1055 nm), ^3^A_2g_ → ^3^T_1g_ (625 nm), and ^3^A_2g_ → ^3^T_1g_(P) (370 nm). The splitting 10*Dq* and Racah parameters were also calculated and the resulting values of 9800 cm^−1^ and 909 cm^−1^, respectively, are consistent with an octahedral geometry for Ni(II). The nephelauxetic parameter value is 0.87 suggesting an ionic character of the metal–ligand bonds [51].

For complex (**3**), the absorption maximum at 700 nm was assigned to d*_xz_*, d*_yz_* → d*_x_*^2^_−*y*_^2^ transition, characteristic of octahedral distorted stereochemistry.

All complexes’ spectra contain two strong bands in UV regions characteristic of π → π* intraligand transitions. These are shifted to lower energies compared with uncoordinated 2,2′-bipyridine bands.

Since the antimicrobial activity was carried out in dimethyl sulfoxide (DMSO) solutions, the complexes’ stability in this media was investigated by UV-Vis spectroscopy. Changes observed over time for each complex solution indicated that all compounds are stable for 48 h (Figure S3).

### 2.3. Biological Activity

In order to highlight potential applications of the obtained compounds, the antimicrobial and biocompatibility features of the obtained compounds have been investigated.

#### 2.3.1. Antimicrobial Activity

In vitro screening of the antimicrobial activities of acrylate complexes (**1**)–(**4**) was performed by the broth microdilution method, in order to establish the minimum inhibitory concentration (MIC) against three microbial strains representative of infections with Gram-positive bacteria (*Staphylocococcus aureus)*, Gram-negative (*Escherichia coli*) bacteria, and fungal (*Candida albicans*) strains (Table 6).

Screening of the antimicrobial activity of complexes (**1**)–(**4**) revealed variable MIC values, ranging between 128 and 1024 μg mL^−1^ for the tested complexes, indicating a moderate to low antimicrobial potential. The antimicrobial activity of the tested complexes was improved by comparison with that of sodium acrylate (NaC_3_H_3_O_2_), as demonstrated by the MIC values which were 4–39 times lower than that obtained for NaC_3_H_3_O_2_. The most susceptible microbial strain was *C. albicans*, towards which all four tested complexes exhibited the same MIC value (128 μg mL^−1^). Regarding the antibacterial effect, the most active compounds were (**3**) and (**4**), displaying an MIC value of 128 μg mL^−1^ against *E. coli* for complex (**3**) and against *S. aureus* for (**4**).

Complex (**1**) revealed moderate antimicrobial activity with an MIC of 256 μg mL^−1^ against the *E. coli* strain. The lowest antibacterial activity was shown by [Ni(2,2′-bipy)(C_3_H_3_O_2_)_2_(H_2_O)] (**2**), exhibiting the highest MIC against both the Gram-positive and Gram-negative bacterial strains (1024 μg mL^−1^). This behavior could be due to the octahedral stereochemistry of Ni(II) in complex (**2**) and its notorious preference for such surrounding, which was shown to have a low antimicrobial activity [26].

#### 2.3.2. Flow Cytometry Analysis

Analysis of the microbial cell populations treated with sub-inhibitory concentrations of the tested compounds allowed us to formulate some hypotheses concerning the putative mechanisms of the antimicrobial activity of the tested compounds. Only the most susceptible strains and the most active compounds (i.e., those exhibiting MIC of 128 µg mL^−1^) have been tested.

Propidium iodide (PI) staining revealed the viability of the cells for all tested combinations, as shown by the negative FL3 signal recorded for the microbial suspensions treated with the tested compounds (Figure S4), proving a microbiostatic rather than microbicidal activity of the tested compounds (Figure S5). This could explain the relatively high MIC values obtained for the respective compounds.

In exchange, the tested compounds were confirmed as potent or moderate efflux pump activity inhibitors (EPI), as revealed by the increased percentage of cells showing cellular uptake of EB, materialized by the occurrence of increased FL2 fluorescence signal typical of EB, directly correlated with the EPI activity of the tested compounds (Figure S6). The most potent EPI activity was noted for (**4**) against *C. albicans* and for (**3**) against *E. coli* (Figure S7).

#### 2.3.3. In Vitro Biocompatibility Assay

**Figure 9:**
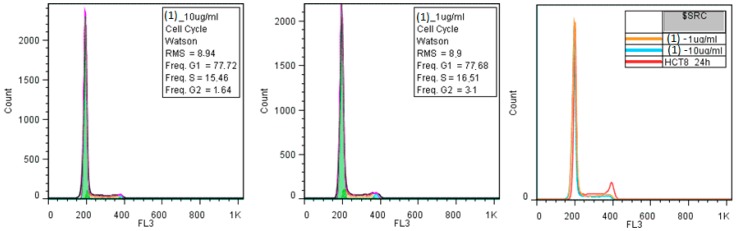

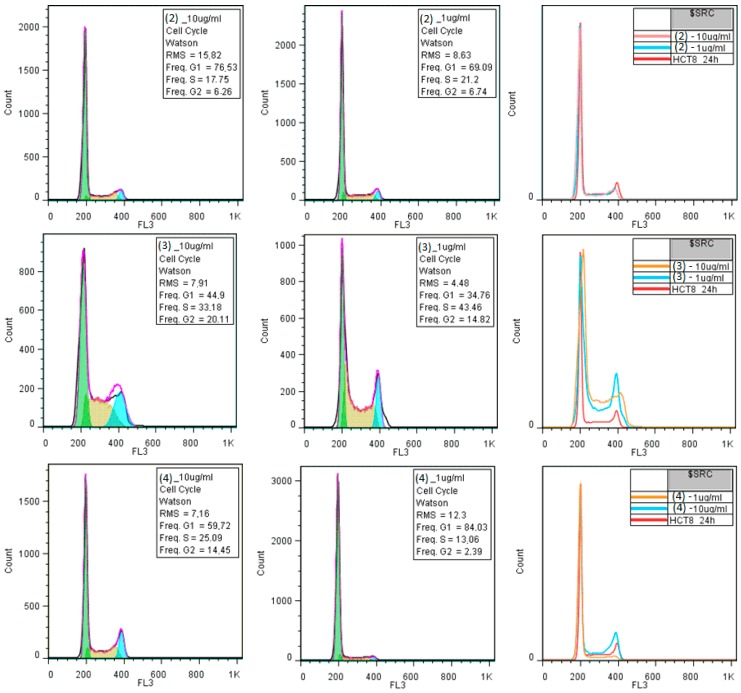
The effects of 10µg/mL (left histograms) or 1 µg/mL (middle histograms) compounds on the HCT8 cell cycle progression. In the right is represented the overlaid histograms of treated and untreated HCT8 cells.

Cell cycle analysis of HCT-8 cells grown in the presence of different concentrations of the tested complexes highlighted that the tested compounds are not toxic, as the area under the G1 phase specific to apoptotic cells was absent (Figure 9) and according trypan blue test (data not shown). Complexes (**1**) and (**2**) induced a slightly increase in the number of cells in the G0/G1 phases, correlated with a decrease in the number of cells in the S and G2/M phases, while complexes (**3**) and (**4**), which also exhibited the most intensive antimicrobial activity, induced an increase in the number of cells in the S and G2/M phases.

## 3. Experimental Section

### 3.1. General Information

All reagents were purchased from Merk Schuchardt OHG (Hohenbrunn, Germany, acrylic acid), Fluka (Saint-Louis, MO, USA, CuCO_3_·Cu(OH)_2_), Acros Organics (Geel, Belgium, NiCO_3_∙2Ni(OH)_2_∙6H_2_O), and Merck (Darmstadt, Germany, MnCO_3_·*x*H_2_O, ZnO and 2,2′-bipyridine); were reagent grade; and were used without further purification.

Chemical analysis of carbon, hydrogen, and nitrogen was performed using a Perkin Elmer PE 2400 analyzer (Perkin Elmer, Waltham, MA, USA). IR spectra were recorded in KBr pellets with a Tensor 37 spectrometer (Bruker, Billerica, MA, USA) in the 400–4000 cm^−1^ range. Diffuse reflectance electronic spectra were recorded at room temperature with a Jasco UV-VIS-NIR V670 spectrometer (Jasco, Easton, MD, USA) in the 200–2000 nm range, using MgO as reference. DMSO solution UV-VIS spectra were recorded on Jasco V530 spectrophotometer in the range of 250–650 nm (Jasco, Easton, MD, USA). X-ray data for complexes (**2**) and (**4**) was collected at room temperature on a STOE IPDS II diffractometer (STOE, Darmstadt, Germany). The structures were solved by direct methods and refined by full-matrix least squares techniques based on F2. The non-H atoms were refined with anisotropic displacement parameters. Calculations were performed using a SHELX-97 crystallographic software package. Data sets for complexes (**1**) and (**3**) were collected with a Nonius Kappa CCD diffractometer (Nonius B. V., Delft, The Netherlands). Programs used: data collection, COLLECT [52]; data reduction, Denzo-SMN [53]; absorption correction, Denzo [54]; structure solution and refinement, SHELX-97 [55,56].

CCDC-1470787 (**1**), -1470788 (**2**), -1470789 (**3**), and -1470790 (**4**) contain the supplementary crystallographic data for this paper. These data can be obtained free of charge from the Cambridge Crystallographic Data Centre via www.ccdc.cam.ac.uk/data_request/cif. The powder diffraction patterns for all complexes (Figures S8–S11, Supplementary Materials) matched well with those simulated from single crystal structure data indicating that bulk samples were isolated as pure phases.

Powder X-ray diffraction (XRD) patterns were recorded using an XRD-7000 diffractometer (Shimadzu, Kyoto, Japan) with Cu Kα radiation (λ = 1.5406 Å, 40 kV, 40 mA) at a step of 0.2° and a scanning speed of 2 degrees min^−1^ in the 5–60 degrees 2θ range.

### 3.2. Synthesis of Complexes

#### 3.2.1. [Mn_3_(2,2′-bipy)_2_(C_3_H_3_O_2_)_6_] (**1**)

A mixture formed from 1.19 g MnCO_3_·*x*H_2_O, 1.4 mL acrylic acid (*ρ* = 1.05 g mL^−1^) and 25 mL distilled water was stirred at room temperature for one hour. The mixture was filtered off in order to eliminate traces of manganese carbonate. To the yellow filtrate a solution composed of 1.25 g of 2,2′-bipyridine and 10 mL ethanol was added and stirred for one hour. The resulting yellow colored solution was left to stand at room temperature, and after one week the obtained yellow crystals were filtered off, washed with ethanol, and air-dried. Yield: 84% (2.53 g), Anal. Calc.: C, 50.51; H, 3.79; N, 6.20; Found: C, 50.41; H, 3.85; N, 6.34%. IR (KBr pellet), cm^−1^: ν(H_2_O), 3441 medium (m); ν(C=C) (acrylate), 1641 very strong (vs); ν_as_(COO), 1597 vs; ν(C=C) + ν(C=N) (bipy), 1540 m; ν_s_(COO), 1362 strong (s), 1341 m; δ(COO), 660 m.

#### 3.2.2. [Ni(2,2′-bipy)(C_3_H_3_O_2_)_2_(H_2_O)] (**2**)

A mixture formed from 0.6 g NiCO_3_∙2Ni(OH)_2_∙6H_2_O, 1 mL acrylic acid (*ρ* = 1.05 g mL^−1^) and 25 mL distilled water was stirred at room temperature for one hour. The reaction mixture was filtered off to eliminate excess carbonate. To the green filtrate a solution containing 0.43 g 2,2′-bipyridine and 10 mL ethanol was added. The blue colored solution was stirred at room temperature for 2 h and then left to evaporate slowly. After one week, light blue crystals were obtained which were filtered off, washed with ethanol, and air dried. Yield: 79% (1.75 g), Anal. Calc.: C, 51.37; H, 4.19; N, 7.52; Found: C, 51.24; H, 4.30; N, 7.47. IR (KBr pellet), cm^−1^: ν(H_2_O), 3378 m, ν(C=C) (acrylate), 1642 s; ν_as_(COO), 1569 vs, 1542 vs; ν(C=C) +ν (C=N) (bipy), 1540 m; ν_s_(COO), 1350 vs, 1371 s; δ(COO), 667 m; *ρ*_w_(H_2_O), 640 m.

#### 3.2.3. [Cu(2,2′-bipy)(C_3_H_3_O_2_)_2_(H_2_O)] (**3**)

A mixture formed from 0.44 g CuCO_3_·Cu(OH)_2_, 0.6 mL acrylic acid (*ρ* = 1.05 g mL^−1^) and 25 mL distilled water was stirred at room temperature for one hour. The reaction mixture was filtered off in order to eliminate excess carbonate. To the blue filtrate a solution containing 0.3 g 2,2′-bipyridine and 10 mL ethanol was added, and the mixture was stirred at room temperature for 2 h. Then, the intense blue solution was allowed to evaporate slowly. After one week blue crystals were obtained which were filtered off, washed with ethanol, and air dried. Yield: 74% (1.12 g), Anal. Calc.: C, 54.50; H, 3.78; N, 6.92; Found: C, 54.43; H, 3.89; N, 6.80. IR (KBr pellet), cm^−1^: ν(H_2_O), 3358 s, ν(C=C)(acrylate), 1635 m; ν_as_(COO), 1571 vs; ν(C=C) + ν(C=N) (bipy), 1540 m; ν_s_(COO), 1360 vs; δ(COO), 661 m; *ρ*_w_(H_2_O), 630 m.

#### 3.2.4. [Zn(2,2′-bipy)(C_3_H_3_O_2_)_2_]*·*H_2_O (**4**)

A suspension containing 0.325 g ZnO, 0.6 mL acrylic acid (*ρ* = 1.05 g mL^−1^) and 25 mL distilled water was stirred at room temperature for one hour. The reaction mixture was filtered off. To the colorless filtrate a solution was added containing 0.62 g 2,2′-bipyridine and 10 mL ethanol. After stirring at room temperature for 2 h, the solution became pale yellow and then was allowed to evaporate slowly at room temperature. After one week pale yellow crystals were p which were filtered off, washed with ethanol, and air dried.

Yield: 92% (1.4 g), Anal. Calc.: C, 50.18; H, 4.03; N, 7.14; Found: C, 50.34; H, 4.22; N, 7.33. IR (KBr pellet), cm^−1^: ν(H_2_O), 3460 s, ν(C=C) (acrylate), 1643 vs; ν_as_(COO), 1551 vs; ν(C=C) + ν(C=N) (bipy), 1535 m; ν_s_(COO), 1368 vs; δ(COO), 689 m.

### 3.3. Biological Assays

#### 3.3.1. Antimicrobial Activity Assay

The following microbial strains were used: Gram-positive (*Bacillus subtilis*, *Staphylococcus aureus*, *Listeria monocytogenes*) bacteria, Gram-negative (*Escherichia coli*, *Salmonella enteritidis*, *Pseudomonas aeruginosa*) bacteria, and one fungal strain (*Candida albicans*). Microbial suspensions of 1.5 × 10^8^ CFU mL^−1^ (0.5 McFarland density) obtained from 15–18 h bacterial cultures developed on solid media were obtained. The compounds were suspended in DMSO to prepare a stock solution of 10 mg mL^−1^ concentration. The quantitative assay of the antimicrobial activity was performed by the liquid medium microdilution method in 96 multi-well plates. Two-fold serial dilutions of the compounds’ solutions (ranging between 4 and 1024 μg mL^−1^) were performed in a 200 μL volume of broth, and each well was seeded with 50 μL of microbial inoculum. Positive controls for microbial cultures (wells containing culture medium seeded with the microbial inoculum) and standard antimicrobials (ciprofloxacin for bacteria and amphotericin B for fungi) were used. The influence of the DMSO solvent was also quantified in a series of wells containing DMSO, diluted accordingly with the dilution scheme. The plates were incubated for 24 h at 37 °C, and the MIC values were considered as the lowest concentration of the tested compound that inhibited the growth of the microbial overnight cultures, as compared to the positive control, revealed by a decreased value of absorbance at 600 nm (Apollo LB 911 ELISA reader, Berthold Technologies GmbH & Co. KG, Bad Wildbad, Germany) [57,58].

#### 3.3.2. Flow Cytometry Assay for the Investigation of Putative Mechanisms of Antimicrobial Activity

Flow cytometry was carried out in order to evaluate the influence of the tested compounds on microbial efflux pump activity. For microbial cell staining, two intercalant fluorochromes with DNA affinity were used: propidium iodide (PI, 10 μg mL^−1^) and ethidium bromide (EB, 5 μg mL^−1^). PI was used for the determination of cellullar viability, as living cells are impermeable to this dye, and EB was used for the detection of bacterial efflux pump activity. Staining procedures were applied to the harvested cells grown in the presence of the tested compounds at the concentration MIC/2. The cells were centrifuged at 13,000 rpm for 3 min, washed 2 times, resuspended in phosphate buffered saline (PBS), stained with 10 μL PI or EB, and incubated for 10 min at 4 °C in the dark. Cells heat-treated for 30 min at 100 °C were used as positive controls and viable cells were used as negative controls. The samples were analyzed with a FACS Calibur instrument (Becton, Dickinson and Company, San Jose, CA, USA) equipped with a 488 nm Argon laser, using a 670 nm long pass filter for the samples stained with PI and a (585 ± 42) nm band pass filter for the samples stained with EB. The log scale was used for all measured parameters. The photomultiplier tube voltages were SSC (side scatter)–550 V, 670 nm long pass filter–480 V, and 585 ± 42 nm band pass filter–500 V. 10,000 events were collected in all runs.

#### 3.3.3. Biocompatibility Assay

HCT-8 (human ileocecal adenocarcinoma) cells were cultivated in RPMI 1640 (Gibco, New York, NY, USA) supplemented with 10% heat-inactivated bovine serum and penicillin/streptomycin, at 37 °C, with 5% CO_2_, in the presence of two concentrations of the tested compounds: 10 μg mL^−1^ and 1 μg mL^−1^, respectively. The 24 h monolayers were harvested, washed with PBS (pH 7.5), fixed in 70% cold ethanol, and maintained overnight at −20 °C. Each sample was washed in PBS, treated with 100 μg mL^−1^ RNase A for 15 min, and stained with 10 μg mL^−1^ PI by incubation at 3 °C for 1 h. After PI staining, events acquisition was performed using an Epics Beckman Coulter flow cytometer (Beckman Coulter, Indianapolis, IN, USA). The obtained data were analyzed using FlowJo software version 7.2.5 (FlowJo LLC, Ashland, OR, USA) and expressed as fractions of cells in different cell cycle phases [59].

## 4. Conclusions

Using the acrylate anion and 2,2′-bipyridine as ligands, four new complexes of Mn(II), Ni(II), Cu(II), and Zn(II) have been prepared and characterized. The trinuclear complex [Mn_3_(2,2′-bipy)_2_(C_3_H_3_O_2_)_6_] (**1**) presents a series of interesting features, such as the linear arrangement of metallic ions and various coordination modes of carboxylate ions (bridge through two oxygen atoms, monoatomic bridge, and chelate). In all complexes, metallic ions adopt an octahedral coordination geometry with different distortion degrees, while acrylato ligands exhibit different coordination modes. The packing diagram of complex (**2**) presents the formation of dimers involving π…π–interactions and additional weak C–H…O hydrogen bonds between the bipyridine unit and the water oxygen atom, while for complex (**3**) the formation of linear chains along the *b*-axis, involving strong O–H…O hydrogen bonds and π…π interactions with alternate distances was shown in the packing diagram. For complex (**4**), there was evidence of weaker π…π stacking interactions in comparison with complex (**3**).

Complexes (**1**)–(**4**) exhibited variable MIC values, ranging from 128 to 1024 μg mL^−1^. All four complexes showed moderate antifungal activity. The most active antibacterial compounds were complex (**3**) against *E. coli* and (**4**) against *S. aureus*. Compounds (**3**) and (**4**) were also potent efflux pumps activity inhibitors (EPI), proving their potential for use in synergistic combinations with antibiotics. They also proved to interfere with the cellular cycle of the tumoral HCT-8 cells by increasing the number of cells found in the S and G2/M phases. Taken together, these results demonstrate the potential of zinc and copper complexes for use in the development of novel antimicrobial and anti-proliferative agents.

## Figures and Tables

**Table 1:** Crystal data and structure refinement for complexes (**1**)–(**4**).

	1	2	3	4
Empirical formula	Mn_3_C_38_H_34_N_4_O_12_	NiC_16_H_16_N_2_O_5_	CuC_16_H_16_N_2_O_5_	ZnC_16_H_14_N_2_O_4_
Formula weight	903.51	375.02	379.85	363.66
Temperature (K)	223(2)	293(2)	223(2)	293(2)
Wavelength λ (Å)	0.71073	0.71073	0.71073	0.71073
Crystal system	triclinic	monoclinic	monoclinic	orthorhombic
Space group	*P*1(2)	*P*2_1_**/***c*(14)	*P*2_1_**/***n*(14)	*Pna*2_1_(33)
a (Å)	10.9974(2)	11.850(2)	21.7184(5)	30.2270(16)
b (Å)	14.0231(3)	16.442(3)	7.0006(2)	7.3771(3)
c (Å)	14.5373(4)	9.386(2)	21.7462(4)	14.9842(7)
α (°)	102.497(1)	90	90	90
β (°)	99.771(1)	113.33(3)	100.914(1)	90
γ (°)	109.161(1)	90	90	90
Volume (Å^3^)	1995.9(1)	1679.2(6)	3246.5(1)	3341.3(3)
Z	2	4	8	8
Reflections collected	30,120	27,530	21,831	52,963
Independent reflections	7889 [R_int_ = 0.039]	3424 [R_int_ = 0.121]	7823 [R_int_ = 0.049]	5992 [R_int_ = 0.0704]
Goodness of fit on F^2^	1.037	1.054	1.029	0.887
*ρ*_calcd_ (g cm^−3^)	1.503	1.483	1.554	1.446
μ (mm^−1^)	1.001	1.183	1.374	1.490
R(Fo), [I > 2σ(I)]	0.041	0.050	0.049	0.031
R_w_ (Fo^2^)	0.114	0.102	0.131	0.063
∆*ρ* (e Å^−3^)	0.62/−0.44	0.43/−0.48	1.12/−0.55	0.21/−0.19

**Table 2:** Selected bond lengths (Å) and angles (°) for [Mn_3_(2,2′-bipy)_2_(C_3_H_3_O_2_)_6_] (**1**).

**Bonds Lengths (Å)**		**Angles (°)**	
Mn1–O3	2.0898(16)	N2–Mn1–N1	72.48(7)
Mn1–O1	2.1026(16)	O4–Mn2–O5	87.99(6)
Mn1–O5	2.1263(15)	O4–Mn2–O9	91.68(6)
Mn1–N2	2.2091(17)	O5–Mn2–O9	91.81(6)
Mn1–N1	2.2670(2)	O4–Mn2–O11	91.25(6)
Mn2–O4	2.1613(15)	O5–Mn2–O11	179.22(6)
Mn2–O5	2.1669(14)	O9–Mn2–O11	88.04(6)
Mn2–O9	2.1692(15)	O4–Mn2–O7	177.46(5)
Mn2–O11	2.1707(14)	O5–Mn–O7	90.18(6)
Mn2–O7	2.1711(15)	O9–Mn2–O7	90.14(6)
Mn2–O2	2.1862(14)	O11–Mn2–O7	90.59(6)
Mn3–O8	2.0813(17)	O4–Mn2–O2	88.73(6)
Mn3–O10	2.0829(16)	O5–Mn2–O2	88.50(6)
Mn3–O11	2.1379(14)	O9–Mn2–O2	179.50(5)
Mn3–N3	2.2152(16)	O11–Mn2–O2	91.66(6)
Mn3–N4	2.2634(18)	O7–Mn2–O2	89.46(6)
**Angles (°)**		O8–Mn3–O10	95.92(7)
O3–Mn1–O1	95.55(7)	O8–Mn3–O11	99.80(6)
O3–Mn1–O5	98.73(7)	O10–Mn3–O11	105.90(6)
O1–Mn1–O5	109.51(6)	O8–Mn3–N3	92.10(6)
O3–Mn1–N2	95.43(6)	O10–Mn3–N3	111.17(6)
O1–Mn1–N2	112.03(6)	O11–Mn3–N3	139.54(6)
O5–Mn1–N2	134.31(6)	O8–Mn3–N4	164.53(6)
O3–Mn1–N1	167.37(7)	O10–Mn3–N4	92.61(7)
O1–Mn1–N1	92.49(7)	O11–Mn3–N4	90.20(6)
O5–Mn1–N1	87.69(7)	N3–Mn3–N4	72.76(6)

**Table 3:** Structural parameters (Å) for trinuclear compounds of the type [Mn_3_(RCOO)_6_(2,2′-bipy)_2_] and carboxylate bridging mode.

Compound	d(Mn_c_‒O_b_)	d(Mn_t_‒O_b_)	d(Mn_t_‒O_d_)	d(Mn_…_Mn)	Carboxylate Bridging Mode	Ref
[Mn_3_((CH_3_)_2_CHCOO)_6_(2,2′-bipy)_2_]	2.177	2.173	2.471	3.489	A	[32]
[Mn_3_(C_6_H_5_CH=CHCOO)_6_(2,2′-bipy)_2_]·H_2_O	2.221	2.218	2.388	3.527	A	[39]
[Mn_3_(CH_2_=CHCOO)_6_(2,2′-bipy)_2_] (**1**)	2.170	2.132	2.722	3.476	B	this paper
[Mn_3_(CH_3_COO)_6_(2,2′-bipy)_2_]	2.202	2.155	2.605	3.614	B	[33]
[Mn_3_(ClCH_2_COO)_6_(2,2′-bipy)_2_]	2.217	2.151	2.611	3.624	B	[38]

**Table 4:** Selected bond lengths (Å) and angles (°) in **2**, **3,** and **4**.

	2 (M = Ni)	3* (M = Cu)	4* (M = Zn)
M–N1	2.075(3)	2.021(3)/2.019(3)	2.100(3)/2.093(2)
M–N2	2.064(3)	2.012(3)/2.009(3)	2.099(2)/2.100(3)
M–O1	2.126(2)	1.991(3)/1.982(3)	1.994(3)/2.012(3)
M–O2	2.147(3)	2.637(3)/2.679(3)	2.490(3)/2.370(4)
M–O3	2.034(2)	1.949(3)/1.957(3)	2.032(3)/1.977(3)
M–O4	-	-/-	2.326(3)/2.563(3)
M–O5w	2.060(3)	2.289(3)/2.281(3)	-/-
N1–M–N2	79.3(1)	80.5(1)/80.4(1)	78.7(1)/78.7(1)

* Two independent molecules were found in the asymmetric unit.

**Table 5:** Selected bond lengths for complexes (**2**)–(**4**) and similar species.

Complex	Bond Lengths (Å)			Reference
	M–N	M–O (Carboxylate)	M–O (Water)	
[Ni(2,2′-bipy)(C_3_H_3_O_2_)_2_(H_2_O)] (**2**)	2.075(3) 2.064(3)	2.034(2)	2.060(3)	this paper
[Ni(2,2′-bipy)(O_2_CMe)_2_(H_2_O)_2_]	2.069(2)	2.079(2)	2.082(2)	[44]
[Ni(dmbipy)(O_2_CMe)_2_(H_2_O)_2_]	2.067(4)	2.077(3)	2.077(3)	
[Cu(2,2′-bipy)(C_3_H_3_O_2_)_2_(H_2_O)] (**3**)	2.021(3)/2.019(3) 2.012(3)/2.009(3)	1.976(4) 2.769(4)	2.289(3)/2.281(3)	this paper
[Cu(2,2′-bipy)_2_(O_2_CPh)]I·0.5H_2_O	1.987(4) 2.192	1.991(3)/1.982(3) 2.637(3)/2.679(3)	-	[45]
[Cu(2,2′-bipy)(C_4_H_5_O_2_)_2_(H_2_O)]	2.023(3) 2.005(3)	1.969(2)/1.926(2)	2.292(2)	[46]
[Cu(2,2′-bipy)_2_(O_2_CMe)]ClO_4_	2.033(19), 1.998(18) 2.017(18), 2.175(19)	1.997(17)	-	[47]
[Zn(2,2′-bipy)(C_3_H_3_O_2_)_2_]·H_2_O (**4**)	2.100(3)/2.093(2) 2.099(2)/2.100(3)	1.994(3)/2.012(3) 2.490(3)/2.370(4)	-	this paper
[Zn(dmbipy)(O_2_CMe)_2_]	2.079(2)	2.058(2) 2.362(3)		[48]

dmbipy: 4,4′-dimethyl-2,2′-bipyridine; C_4_H_5_O_2_: methacrylate ion.

**Table 6:** Minimum inhibitory concentration (μg mL^−1^) values recorded for complexes (**1**)–(**4**), sodium acrylate, and standard antibiotics.

	Strains	Gram-Positive Bacterial Strain	Gram-Negative Bacterial Strain	Fungal Strain
Compound		*S. aureus* ATCC 25923	*E. coli* ATCC 25922	*C. albicans* ATCC 10231
(**1**)		512	256	128
(**2**)		1024	1024	128
(**3**)		512	128	128
(**4**)		128	1024	128
**NaC_3_H_3_O_2_**		5000	5000	5000
CIP *		0.5	0.05	
AMB *				0.5

* CIP: ciprofloxacin; AMB: amphotericin B.

## Supplementary Materials

The supplementary materials are available online.

## References

[1] Oldham C., Wilkinson G., Gillard R.D., McCleverty J.A. (1987). Carboxylates, squarates and related species. Comprehensive Coordination Chemistry.

[2] Ye B.-H., Tong M.-L., Chen X.-M. (2005). Metal-organic molecular architectures with 2,2′-bipyridyl-like and carboxylate ligands. Coord. Chem. Rev..

[3] Konar S., Zangrando E., Drew M.G.B., Ribas J., Chaudhuri N.R. (2004). Synthesis, structural analysis, and magnetic behaviour of three fumarate bridged coordination polymers: Five-fold interpenetrated diamond-like net of Ni^II^, sheets of Ni^II^ and Co^II^. Dalton Trans..

[4] Ying S.-M., Mao J.-G., Sun Y.-Q., Zheng H.-Y., Dong Z.-C. (2003). Syntheses and crystal structures of three open-frameworks of metal succinates containing a 4,4′-bipyridine ligand. Polyhedron.

[5] Zhou L.-J., Luan X.-J., Wang Y.-Y., Lee G.-H., Shi Q.-Z., Peng S.-M. (2006). Supramolecular complexes constructed with carboxylate Cu(II) and 2-(2-pyridyl)-benzimidazole via hydrogen bonding. J. Coord. Chem..

[6] Devereux M., McCann M., Leon V., Geraghty M., McKee V., Wikaira J. (2000). Synthesis and fungitoxic activity of manganese(II) complexes of fumaric acid: X-ray crystal structures of [Mn(fum)(bipy)(H_2_O)] and [Mn(Phen)_2_(H_2_O)_2_](fum)·4H_2_O (fumH_2_ = fumaric acid; bipy = 2,2′-bipyridine; phen = 1,10-phenanthroline). Polyhedron.

[7] Saidul Islam M., Hossain M.B., Reza M.Y. (2003). Antimicrobial studies of mixed ligand transition metal complexes of maleic acid and heterocyclic amine bases. J. Med. Sci..

[8] Jaguar-Grodzinski J. (1999). Biomedical application of functional polymers. React. Funct. Polym..

[9] Vehof J.W.M., Fisher J.P., Dean D., van der Waerden J.-P.C.M., Spauwen P.H.M., Jansen J.A., Mikos A.G. (2002). Bone formation in transforming growth factor β-1-coated porous poly(propylene fumarate) scaffolds. J. Biomed. Mater. Res..

[10] Badea M., Olar R., Marinescu D., Vasile G. (2006). Thermal behavior of some new complexes bearing ligands with polymerisable groups. J. Therm. Anal. Calorim..

[11] Badea M., Olar R., Marinescu D., Lazar V., Chifiriuc C., Vasile G. (2009). Thermal behaviour of new biological active cadmium mixed ligands complexes. J. Therm. Anal. Calorim..

[12] Liu P., Wang Y.-Y., Li D.-S., Luan X.-J., Gao S., Shi Q.-Z. (2005). One-dimensional polymers constructed with binuclear copper(II) α,β-unsaturated carboxylates bridged by 4,4′-bipyridine. Chin. J. Chem..

[13] Wang X.-W., Chen F.-P., Chen L., Chen J.-Z. (2007). Crystal structure and fluorescence properties of a new ternary binuclear complex: Sm_2_(C_3_H_3_O_2_)_6_(phen)_2_. Z. Naturforsch. B.

[14] Cai T.J., Jiang W.-J., Peng Z.-S., Long Y.-F., Deng Q. (2006). Crystal structure of chloro(acrylato-*O*,*O*′)bis(1,10-phenanthroline-*N*,-*N*′)-cadmium(II), CdCl(C_3_H_3_O_2_)(C_12_H_8_N_2_)_2_. Z. Kristallogr. New Cryst. Struct..

[15] Badea M., Olar R., Marinescu D., Vasile G. (2005). Some new acrylate complexes as a criterion in their selection for further co-polymerization reaction. J. Therm. Anal. Calorim..

[16] Badea M., Olar R., Marinescu D., Vasile G. (2008). Thermal stability of new complexes bearing both acrylate and aliphatic amine as ligands. J. Therm. Anal. Calorim..

[17] Wang Y.-Y., Shi Q., Shi Q.-Z., Gao Y.-C., Zhou Z.-Y. (1999). Syntheses, characterization and crystal structure of copper(II) α,β-unsaturated carboxylate complexes with imidazole. Polyhedron.

[18] Wang Y.-Y., Zhou L.J., Shi Q., Shi Q.-Z. (2002). Novel trinuclear copper(II) complexes with α,β-unsaturated carboxylates and imidazole. Trans. Met. Chem..

[19] Vlaicu I.D., Olar R., Marinescu D., Lazar V., Badea M. (2013). Physico-chemical and thermal characterisation of new Co(II) complexes with pyrazole derivatives. J. Therm. Anal. Calorim..

[20] Wu H.-L., Gao Y.-C. (2004). Synthesis, crystal structure and characterization of zinc(II) complexes with the tripod ligand tris(2-benzimidazolylmethyl)amine and α,β-unsaturated carboxylates. Trans. Met. Chem..

[21] Wu H.-L., Gao Y.-C. (2006). Synthesis, crystal structure and properties of manganese(II) complexes with the tripod ligand tris(2-benzimidazylmethyl)amine and α,β-unsaturated carboxylates. J. Coord. Chem..

[22] Badea M., Vlaicu I.D., Olar R., Constand M., Bleotu C., Chifiriuc M.C., Marutescu L., Lazar V., Grecu M.N., Marinescu D. (2014). Thermal behaviour and characterisation of new biologically active Cu(II) complexes with benzimidazole as main ligand. J. Therm. Anal. Calorim..

[23] Vlaicu I.D., Constand M., Olar R., Marinescu D., Grecu M.N., Lazar V., Chifiriuc M.C., Badea M. (2013). Thermal stability of new biologic active copper(II) complexes with 5,6-dimethylbenzimidazole. J. Therm. Anal. Calorim..

[24] Olar R., Vlaicu I.D., Chifiriuc M.C., Bleotu C., Stanică N., Vasile Scăeţeanu G., Silvestro L., Dulea C., Badea M. (2017). Synthesis, thermal analysis and biological characterisation of some new nickel (II) complexes with unsaturated carboxylates and heterocyclic N-donor ligands. J. Therm. Anal. Calorim..

[25] Badea M., Olar R., Marinescu D., Vasile G. (2008). Thermal behavior of some new triazole derivative complexes. J. Therm. Anal. Calorim..

[26] Wang Y.-Y., Liu P., Qian S., Gao Y., Shi Q.-Z. (2001). Syntheses, characterization, crystal structure and magnetic properties of copper(II) α,β-unsaturated carboxylate complexes with trimethyl phosphate. Chin. Sci. Bull..

[27] Wang Y.-Y., Shi Q., Shi Q.-Z., Gao Y.-C., Zhou Z. (1999). Synthesis, thermal decomposition and crystal structure of copper(II) α,β-unsaturated carboxylate with urea. Chin. Sci. Bull..

[28] Jarrah A., Shafaghat A., Dadkhah M. (2011). Antibacterial activity of Cu(II) and Co(II) complexes of 3,4-dihydroxybenzeneacrylic acid against the pathogen, nonpathogenic bacteria and sonochemical synthesis of nanoscale mixed-ligand EDA coordination for preparation of CoC1_2_·6H_2_O nanoparticle. World Appl. Sci. J..

[29] Jarrah A., Shafaghat A., Dadkhah M. (2011). Synthesis, characterization and anti-tumor activity of Cu(II) and Co(II) complexes of 3-(3,4-dihydroxy benzene acrylic acid). World Appl. Sci. J..

[30] Wang Y.-Y., Shi Q., Shi Q.-Z., Gao Y.-C., Hu X. (2000). Molecular structure, characterization and magnetic properties of novel mixed-valence copper(I,II) α,β-unsaturated carboxylate complexes with triphenylphosphine and methanol ligands. Polyhedron.

[31] Limban C., Chifiriuc C., Grumezescu A.M. (2013). Thiourea Derivatives as Antimicrobials.

[32] Baca S., Sevryugina Y., Clerac R., Malaestean I., Gerbeleu N., Petrukhina M. (2005). Linear trinuclear manganese(II) complexes: Crystal structures and magnetic properties. Inorg. Chem. Commun..

[33] Menage S., Vitols S.E., Bergerat P., Codjovi E., Kahn O., Girerd J.-J., Guillot M., Solans X., Calvet T. (1991). Structure of the Linear Trinuclear Complex Mn^II^_3_(CH_3_CO_2_)_6_(bpy)_2_. Determination of the J Electron-Exchange Parameter through Magnetic Susceptibility and High-Field Magnetization Measurements. Inorg. Chem..

[34] Tsuneyoshi K., Kobayashi H., Miyamac H. (1993). Structure of hexa-μ-acetato-1κ^3^*O*:2κ^3^*O*′;2κ^3^*O*:3κ^3^*O*′-bis(1,10-phenanthroline)-1κ^2^*N*^1^,*N*^10^;2κ^2^*N*^1^,*N*^10^-trimanganese(II). Acta Cryst. C.

[35] Rardin R.L., Poganiuch P., Bino A., Goldberg D.P., Tolman W.B., Liu S., Lippard S.J. (1992). Synthesis and Characterization of Trinuclear Iron(II) and Manganese(II) Carboxylate Complexes: Structural Trends in Low Valent Iron and Manganese Carboxylates. J. Am. Chem. Soc..

[36] Tangoulis V., Malamatari D.A., Soulti K., Stergiou V., Raptopoulou C.P., Terzis A., Kabanos T.A., Kessissoglou D.P. (1996). Manganese(II/II/II) and Manganese(III/II/III) Trinuclear Compounds. Structure and Solid and Solution Behavior. Inorg. Chem..

[37] Wortmann R., Flörke U., Sarkar B., Umamaheshwari V., Gescheidt G., Herres-Pawlis S., Henkel G. (2011). Synthesis and characterisation of novel (Guanidine) manganese complexes and their application in the epoxidation of 1-octene. Eur. J. Inorg. Chem..

[38] Fernandez G., Corbella M., Corbella J., Mahia J., Maestro M. (2002). Polynuclear Mn^II^ complexes with chloroacetate bridge–Syntheses, structure, and magnetic properties. Eur. J. Inorg. Chem..

[39] Chen Y., Wang X.-W., Hu B., Chen F.-P., Chen J.-Z., Chen L. (2007). Synthesis, characterization and crystal structure of a new linear trinuclear manganese(II) complex, [Mn_3_(PhCH=CHCO_2_)_6_(BPY)_2_]·H_2_O (BPY=2,2-bipyridine). J. Coord. Chem..

[40] Kang B., Kim M., Lee J., Do Y., Chang S. (2006). Trimanganese complexes bearing bidentate nitrogen ligands as a highly efficient catalyst precursor in the epoxidation of alkenes. J. Org. Chem..

[41] Kloskowski M., Pursche D., Hoffmann R.-D., Pöttgen R., Läge M., Hammerschmidt A., Glaser T., Krebs B. (2007). Novel trinuclear Mn^II^/Mn^II^/Mn^II^ complexes–Crystal structures and catalytic properties. Z. Anorg. Allg. Chem..

[42] Gómez V., Corbella M. (2009). Versatility in the Coordination Modes of *n*-Chlorobenzoato Ligands: Synthesis, Structure and Magnetic Properties of Three Types of Polynuclear Mn^II^ Compounds. Eur. J. Inorg. Chem..

[43] Baca S., Malaestean I., Keene Y., Adams H., Ward M., Hauser J., Neels A., Decurtins S. (2008). One-dimensional manganese coordination polymers composed of polynuclear cluster blocks and polypyridyl linkers: Structures and properties. Inorg. Chem..

[44] Ye B.-H., Chen X.-M., Xue G.-Q., Ji L.N. (1998). Mononuclear nickel complexes assembled into two-dimensional networks via hydrogen bonds and π–π stacking interactions. J. Chem. Soc. Dalton Trans..

[45] Tang C.-M., Deng G.-H. (2008). Bis(2,2′-bipyridine-*k*^2^*N*,*N*′)(4-methyl-benzoato-*k*^2^*O*,*O*′)copper(II) iodide hemihydrate. Acta Cryst. E.

[46] Yici G., Yaoyu W., Ying Z., Qizhen S. (1991). Crystal structure of bis(α-methacrylato)-2,2′-bipyridine-monohydrate copper. Polyhedron.

[47] Jayamani A., Sengottuvelan N., Kang S.K., Kim Y.-I. (2015). Mono- and binuclear copper(II) complexes of the bipyridine ligand: Structural, electrochemical and biological studies. Polyhedron.

[48] Harvey M.A., Suarez S.A., Ibanez A., Doctorovich F., Baggio R. (2012). Bis(acetato-*k*^2^*O*,*O*′)(4,4′-dimethyl-2,2′-bipyridine-*k*^2^*N*,*N*′)zinc. Acta Cryst. E.

[49] Deacon G.B., Philips J.R. (1980). Relationships between the carbon-oxygen stretching frequencies of carboxylato complexes and the type of carboxylate coordination. Coord. Chem. Rev..

[50] Nakamoto K. (2009). Infrared and Raman Spectra of Inorganic and Coordination Compounds, Part B, Applications in Coordination, Organometallic, and Bioinorganic Chemistry.

[51] Lever A.B.P. (1984). Inorganic Electronic Spectroscopy.

[52] Hooft R.W.W. (1998). COLLECT, Program for Collecting Data on CCD Area Detectors.

[53] Otwinowski Z., Minor W. (1997). Processing of X-ray diffraction data collected in oscillation mode. Methods Enzymol..

[54] Otwinowski Z., Borek D., Majewski W., Minor W. (2003). Multiparametric scaling of diffraction intensities. Acta Crystallogr. A.

[55] Sheldrick G.M. (1990). Phase annealing in *SHELX*-90: Direct methods for larger structures. Acta Crystallogr. A.

[56] Sheldrick G.M. (2008). A short history of *SHELX*. Acta Crystallogr. A.

[57] Stecoza C.E., Cǎproiu M.T., Drǎghici C., Chifiriuc M.C., Drǎcea N.O. (2009). Synthesis, characterization and antimicrobial activity evaluation of some new derivatives of 6,11-dihydrodibenzo[*b*,*e*]thiepin 5,5-dioxide. Rev. Chim..

[58] Limban C., Chifiriuc M.C. (2011). Antibacterial activity of new dibenzoxepinone oximes with fluorine and trifluoromethyl group substituents. Int. J. Mol. Sci..

[59] Balaure P.C., Andronescu E., Grumezescu A.M., Ficai A., Huang K.S., Yang C.H., Chifiriuc C.M., Lin Y.S. (2013). Fabrication, characterization and in vitro profile based interaction with eukaryotic and prokaryotic cells of alginate-chitosan-silica biocomposite. Int. J. Pharm..

