# Supplementary material for: Synthesis, Structural Characterization, Antimicrobial Activity, and In Vitro Biocompatibility of New Unsaturated Carboxylate Complexes with 2,2′-Bipyridine
Source: Molecules. 2018 Jan 12;23(1):157. doi: 10.3390/molecules23010157 (PMC6017882; doi:10.3390/molecules23010157)
Supplement: Supplementary file 1 [file molecules-23-00157-s001.pdf]

Supplementary materials: Synthesis, structural characterization, antimicrobial activity and in vitro biocompatibility of new unsaturated carboxylate complexes with 2,2′-bipyridine

Gina Vasile Scăețeanu, Mariana Carmen Chifiriuc, Coralia Bleotu, Crina Kamerzan, Luminița Măruțescu, Constantin G. Daniliuc, Cătălin Maxim, Larisa Calu, Rodica Olar and Mihaela Badea

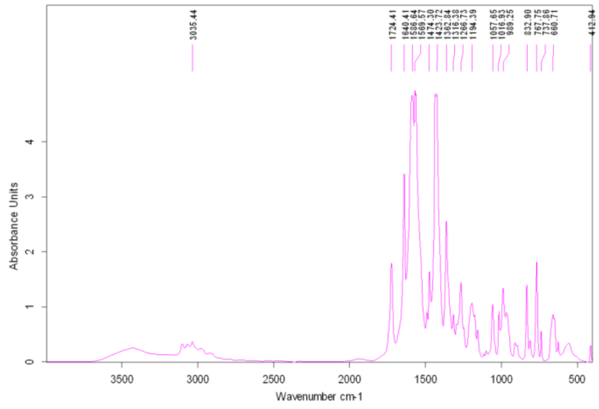

(1)

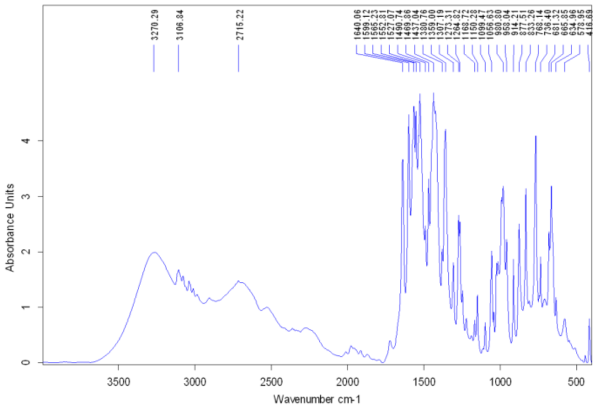

(2)

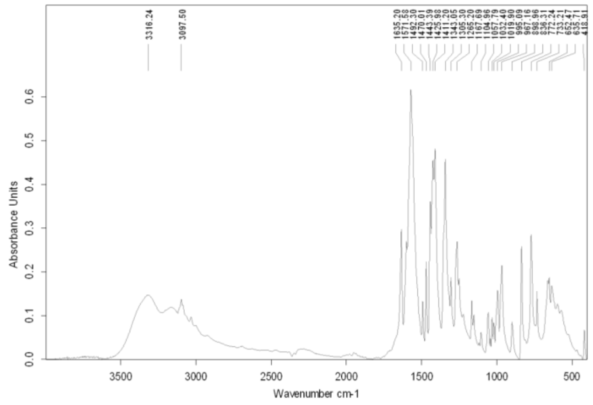

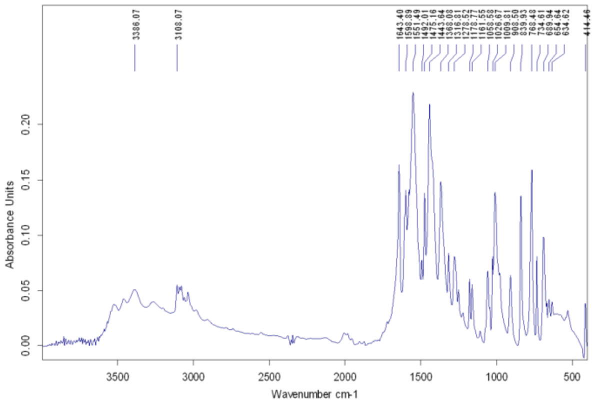

(3)

(4)

Figure S1. IR spectra of complexes (**1**)-(**4**).

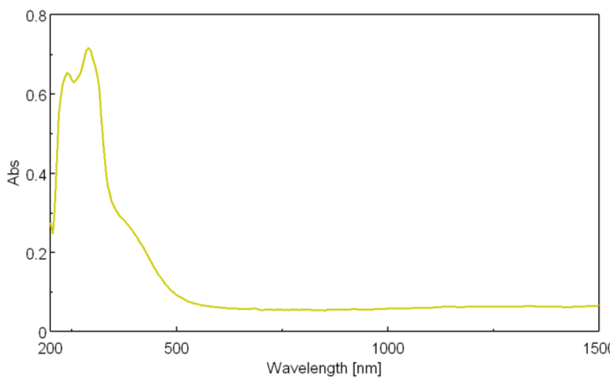

(1)

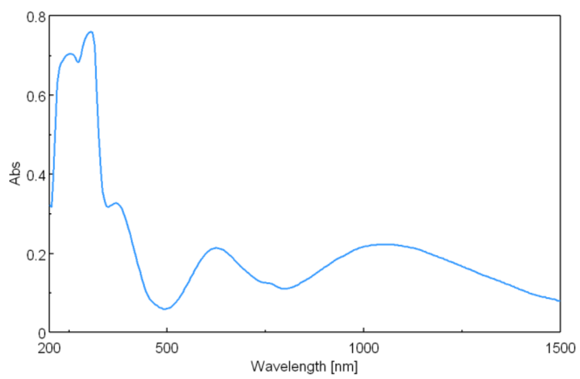

(2)

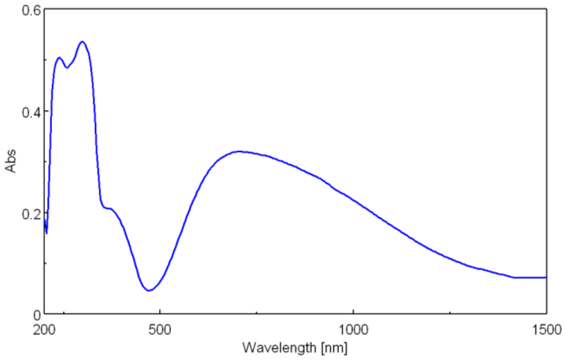

(**3**)

Figure S2. UV-Vis-NIR spectra of complexes (**1**)-(**3**).

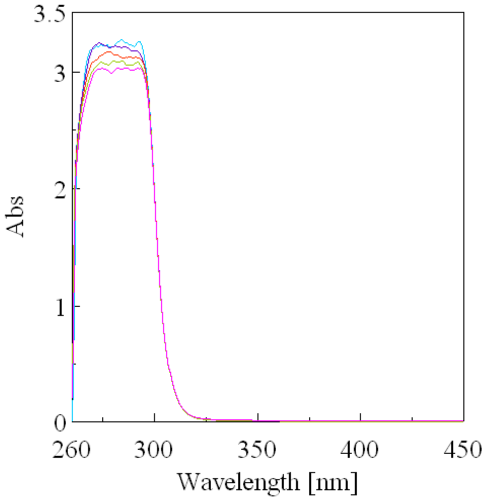

(**1**)

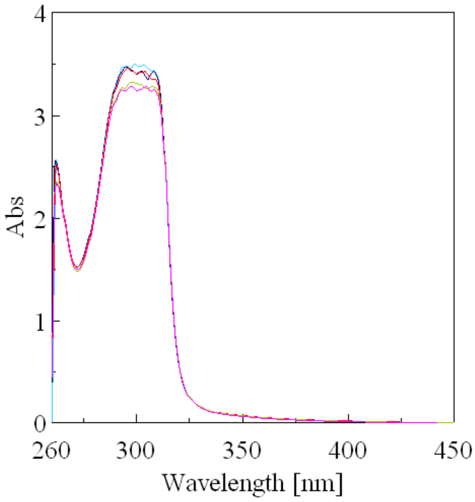

(**2**)

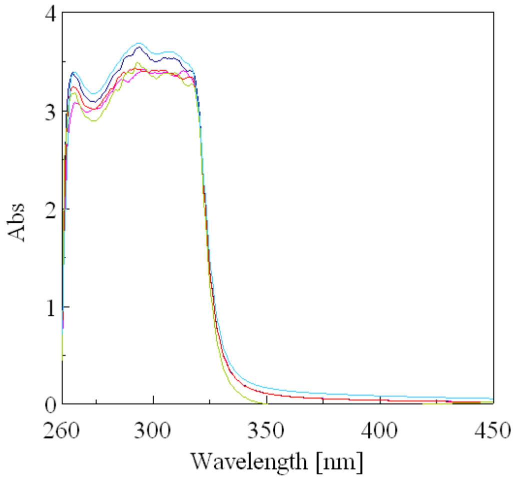

(**3**)

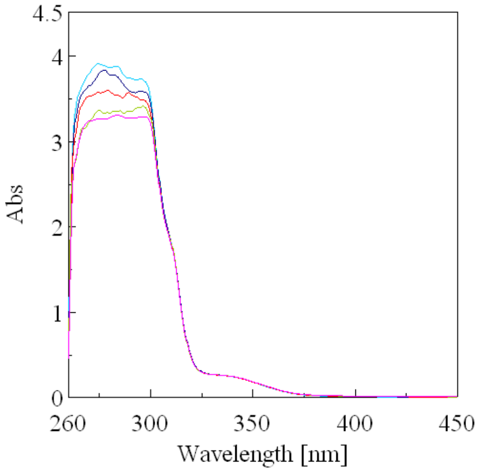

(**4**)

Figure S3. UV-Vis spectra in DMSO solution of complexes (**1**)-(**4**): (blue – 0 min, purple – 6 h, red – 12 h, green – 24 h, magenta -48h).

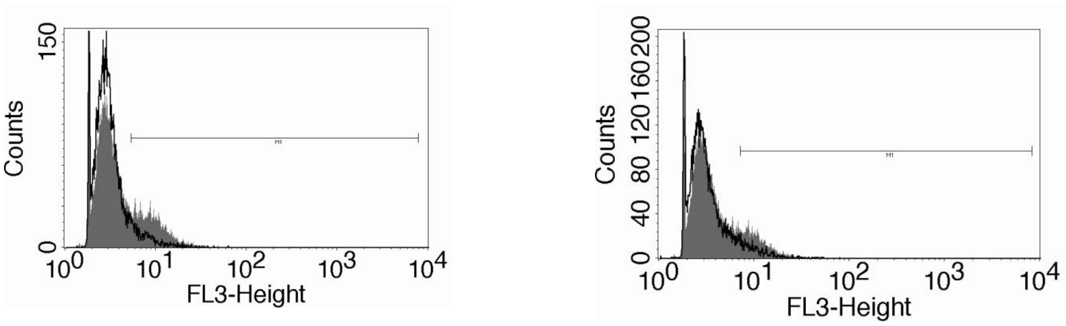

E. coli (**3**) *C. albicans* (**4**)

Figure S4. Typical fluorescence histograms indicating the negative PI fluorescence signal (left shifted) correlated with the viability of the microbial cells in the presence of the tested compounds.

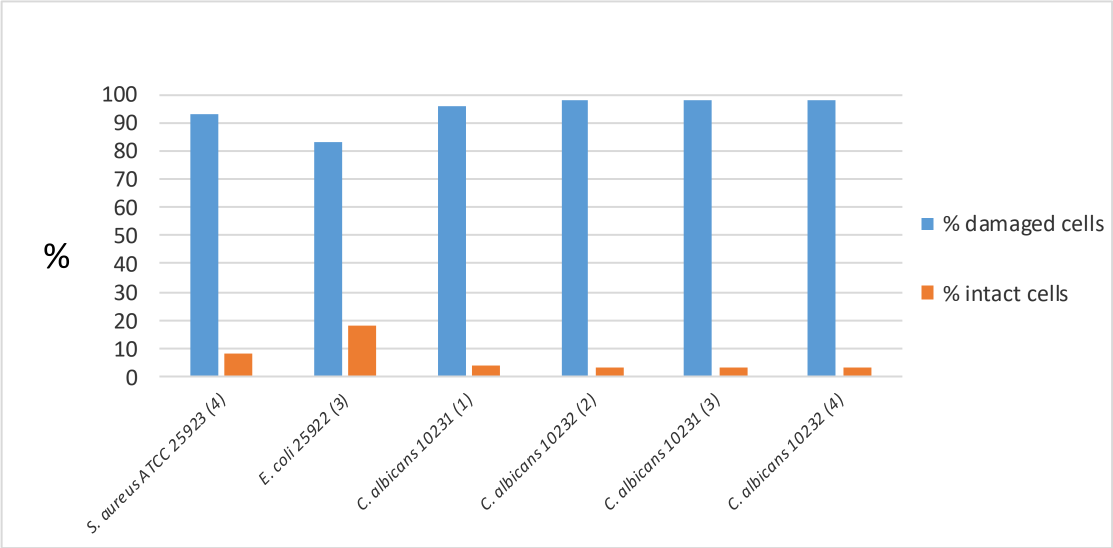

Figure S5. The effect of complexes (**1**)-(**4**) on cellular membrane permeability assessed by flow cytometry.

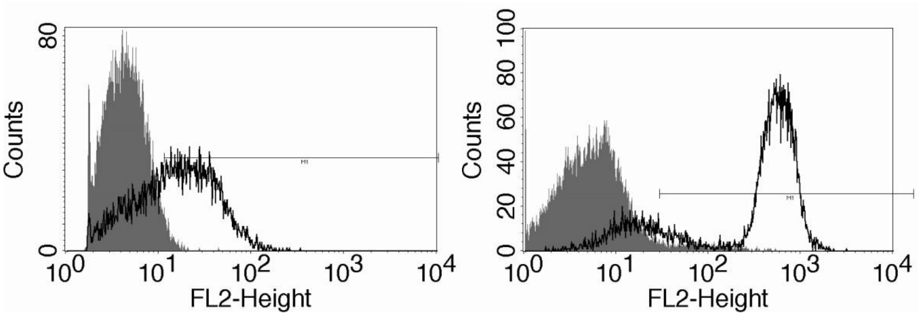

E. coli (**3**) *C. albicans* (**4**)

Figure S6. Typical fluorescence histograms indicating the positive EB fluorescence signal (right shifted) correlated with the efflux pumps inhibitory effect of the tested compounds.

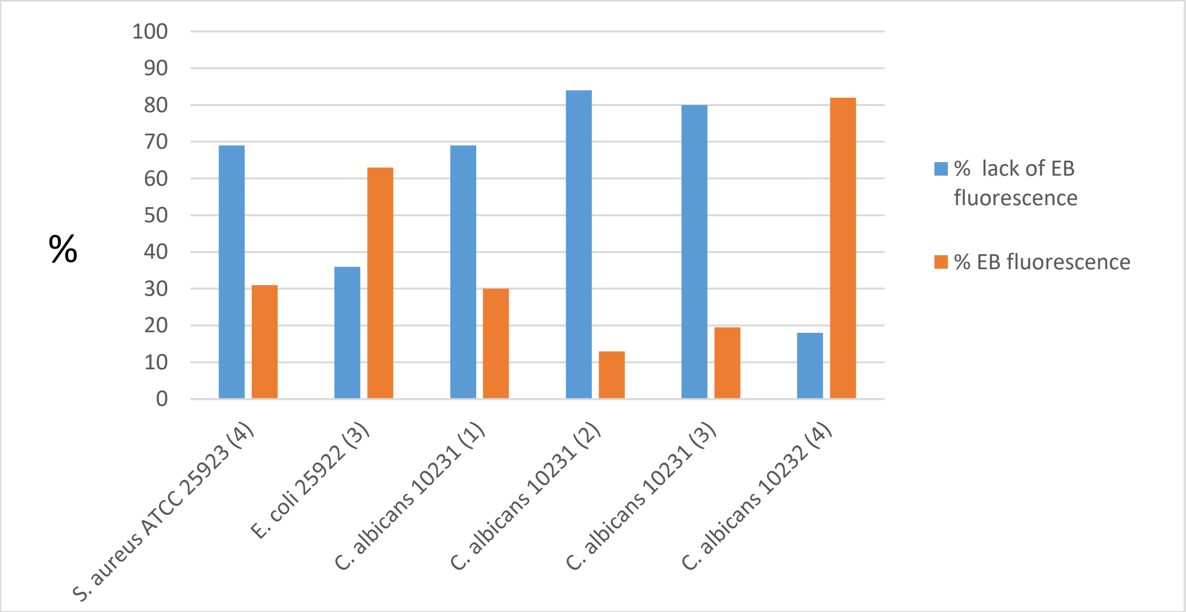

Figure S7. The effect of complexes (**1**)-(**4**) on the activity of efflux pumps assessed by flow cytometry.

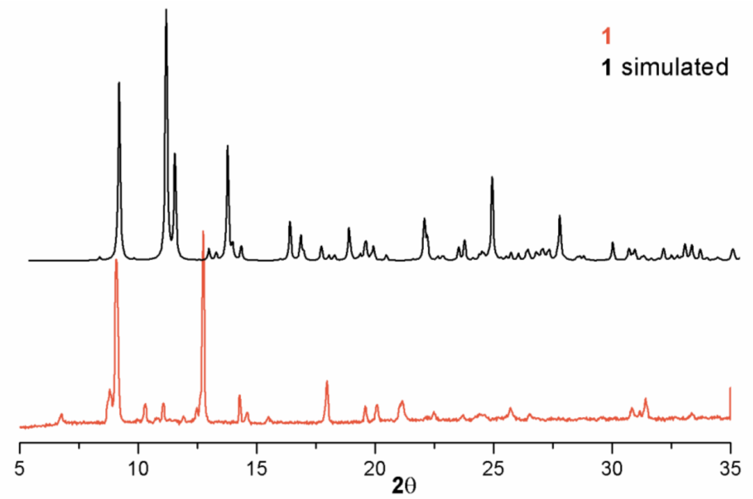

Figure S8. XRPD patterns for compound **1** (in orange) shown in comparison with the XRPD pattern of **1** simulated from SC-XRD data (in black)

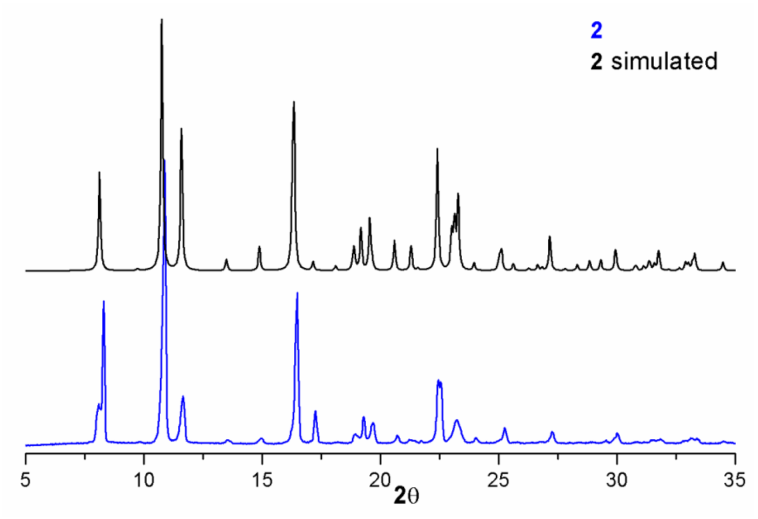

Figure S9. XRPD patterns for compound **2** (in blue) shown in comparison with the XRPD pattern of **2** simulated from SC-XRD data (in black)

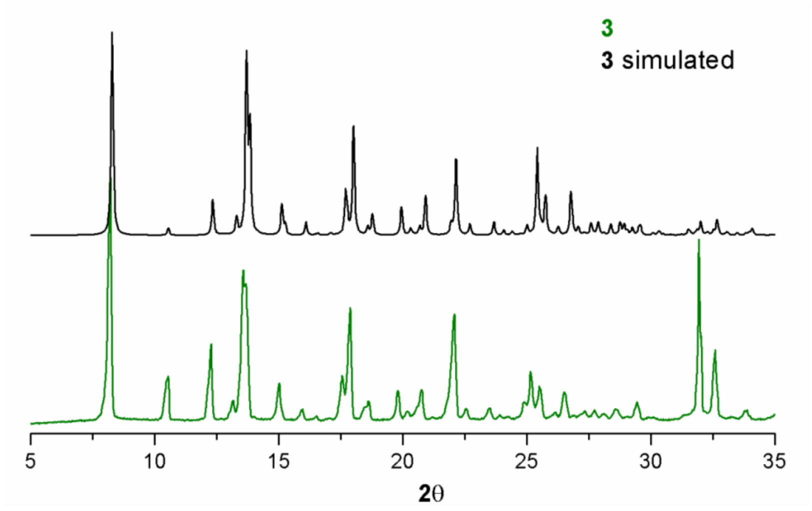

Figure S10. XRPD patterns for compound **3** (in green) shown in comparison with the XRPD pattern of **3** simulated from SC-XRD data (in black)

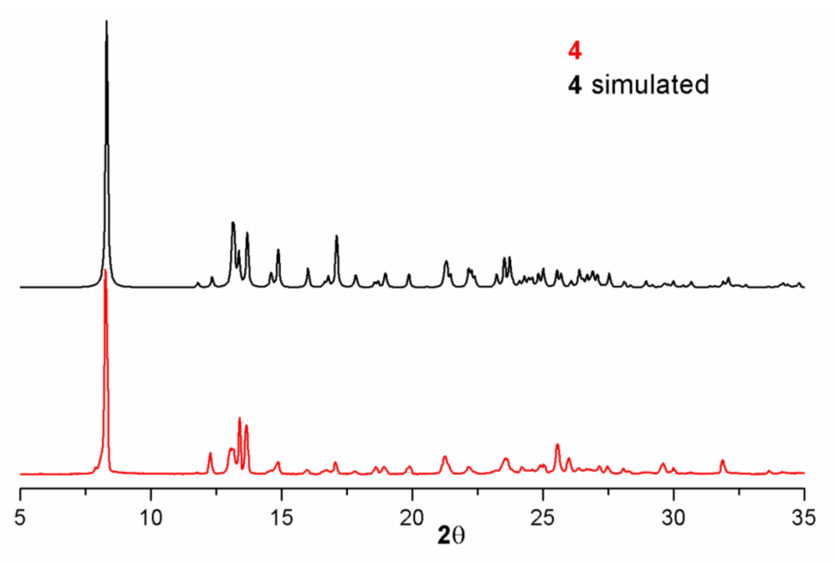

Figure S11. XRPD patterns for compound **4** (in red) shown in comparison with the XRPD pattern of **4** simulated from SC-XRD data (in black)
